# Novel pharmacotherapies for opioid use disorder and opioid withdrawal

**DOI:** 10.3389/fpsyt.2026.1909149

**Published:** 2026-09-03

**Authors:** Mary R. Shen, Kwadwo Owusu-Boaitey, Zackari D. Murphy, Alex N. Rains, Philip R. Wang, Dennis W. Zhou, Joji Suzuki

**Affiliations:** Department of Psychiatry, Brigham and Women’s Hospital, Harvard Medical School, Boston, MA, United States

**Keywords:** addiction, cannabinoid, drug, GLP-1, ketamine, opioid, psychedelics, withdrawal

## Abstract

**Introduction:**

Opioid use disorder (OUD) remains a major public health crisis despite evidence-based medications, including methadone, buprenorphine, and naltrexone. Persistent challenges with treatment retention, access, stigma, and incomplete response highlight the need for adjunctive pharmacotherapies targeting neurobiological systems beyond the mu-opioid receptor.

**Methods:**

We conducted a narrative review of emerging pharmacologic approaches for OUD and opioid withdrawal syndrome, focusing on ketamine and NMDA receptor antagonists, cannabinoids, psychedelics, and incretin-based therapies, including glucagon-like peptide-1 receptor agonists and dual glucose-dependent insulinotropic polypeptide/glucagon-like peptide-1 receptor agonists.

**Results:**

Ketamine has preliminary randomized evidence suggesting potential effects on abstinence, withdrawal, craving, and psychotherapy augmentation. Cannabidiol may reduce cue-induced craving and anxiety, whereas dronabinol may modestly suppress opioid withdrawal symptoms. Psychedelic research, particularly involving ibogaine, shows observational signals for withdrawal reduction and abstinence but is limited by safety concerns, regulatory barriers, and sparse controlled evidence. Incretin-based therapies have generated strong observational signals linking GLP-1–based treatment to reduced overdose and OUD-related outcomes, though prospective trials remain limited.

**Discussion:**

Across these therapeutic classes, convergent mechanisms include modulation of mesolimbic reward circuitry, cue-reactivity, stress responsivity, neuroplasticity, and cognitive flexibility. Future studies should prioritize rigorous blinding assessment, active comparators, objective endpoints, standardized cue-reactivity measures, diverse samples, and careful safety monitoring. Regulatory policies should facilitate the development and implementation of future research in novel therapies for OUD.

## Introduction

Opioid use disorder (OUD) and opioid withdrawal syndrome (OWS) remain a public health crisis in the U.S. and globally with close to 80,000 overdose deaths in 2023 (1, 2), despite evidence-based medications for opioid use disorder (MOUD). An urgent need exists for new pharmacotherapies to treat OUD and opioid-related morbidity and mortality.

Current FDA-approved medications for OUD include methadone, buprenorphine, and naltrexone, which have full agonist, partial agonist, and antagonist action on the mu-opioid receptor, respectively (3). Despite strong evidence demonstrating that methadone and buprenorphine reduce mortality (4, 5) and naltrexone reduces morbidity (6), utilization is limited by early discontinuation, access barriers, and associated stigma (7, 8). Approximately 40-60% of patients newly initiating OUD treatment discontinue by 6 months, with the highest rates occurring within the first 3 months (9). Identifying novel targets beyond the µ-opioid-receptor, facilitated by a broader view of addiction neurobiology, may, therefore, be important for improving existing OUD treatments and establishing new treatment paradigms.

Existing neurobiological models of addiction strongly tie anatomical brain regions and circuits to various features of addiction. For example, drug reward and incentive salience are known to involve activation of mesolimbic reward regions (i.e., nucleus accumbens, dorsal striatum), receiving input from dopaminergic projections of the ventral tegmental area and excitatory input from the prefrontal cortex and amygdala (10). Relatedly, executive control in addiction involves activation of the prefrontal cortex through glutamatergic projections to the striatum with reciprocal feedback (11). These circuits depend on diverse neurotransmitters and cellular processes beyond opioid signaling and growing interest has focused on pharmacotherapies that target these broader neural systems.

In this review, we highlight four emerging pharmacologic approaches for OUD: Glucagon-like peptide-1 receptor agonists (GLP-1 RAs), ketamine, cannabinoids, and psychedelics, summarizing their mechanistic rationale, the current state of clinical evidence, and limitations to the research.

## Methods

For this narrative review, we conducted structured journal database searches (i.e., PubMed, Google Scholar) to identify English language, peer-reviewed human studies addressing opioid use disorder, opioid withdrawal, and four emerging therapeutic areas: ketamine, cannabinoids, psychedelics, and GLP-1 receptor agonists. Searches combined opioid-related keywords and MeSH terms with medication- and class-specific terms for each therapeutic category. We supplemented these searches by reviewing reference lists of relevant articles and incorporating studies identified through the senior author’s expertise in addiction medicine. Given the early and rapidly evolving nature of this literature, we aimed to provide a focused narrative overview of each approach, with attention to proposed mechanisms of action, the current evidence base, relevant policy considerations, and implications for clinical practice and future research.

## Results

### Ketamine and NMDA antagonists

Ketamine is a non-competitive antagonist of glutamatergic N-methyl-D-aspartate (NMDA) receptors. Upon binding, ketamine decreases inhibitory tone, triggering rapid glutamate release from pyramidal neurons with further downstream effects thought to promote neuroplasticity, cognitive flexibility, and learning capacity (12). These mechanisms may be particularly relevant to addiction, including opioid use disorder, in which executive functioning, reward learning, and cognitive flexibility are disrupted due to changes in prefrontal, limbic, and hippocampal circuits. By downregulating cue-responsive mesolimbic circuits and improving neuroplasticity, ketamine may serve as an effective adjunct to psychosocial therapies for OUD, such as contingency management and cognitive-behavioral therapy (13). In addition, ketamine and its metabolites may act as positive allosteric modulators of µ- and delta-opioid-receptors, and emerging evidence suggests that ketamine may bind and activate opioid receptors at orthosteric sites directly. There is ongoing debate around ketamine’s opioid receptor interactions and the extent to which these contribute to ketamine’s utility in easing withdrawal and initiating MOUD (14–16). Together, these pathways provide a mechanistic rationale for investigating ketamine as a potential adjunctive treatment for OUD and OWS.

Ketamine is available in multiple formulations that differ in pharmacokinetics and bioavailability. Intravenous (IV) racemic ketamine, containing 1:1 mixture of R- and S-enantiomers, is commonly used in addiction research for its near complete bioavailability, precise dosing, and rapid onset. The S-enantiomer, esketamine, has an FDA-approved intranasal formulation (Spravato) for treatment-resistant depression with estimated 45% bioavailability. Oral and sublingual formulations show the lowest bioavailability (30%) due to first-pass hepatic metabolism (17, 18). Psychiatric protocols generally use lower doses, activating glutamatergic effects while dissociative effects remain short-lived (19).

Krupitsky et al. demonstrated dose-dependent efficacy of ketamine-assisted psychotherapy for heroin use disorder in two foundational trials: a 2002 study showing greater than one-year abstinence rates with higher ketamine dosing (2.0 vs. 0.2 mg/kg) (20) and a 2007 study showing that abstinence and efficacy increased with the number of treatment sessions (21). In the only RCT examining ketamine in the initiation of MOUD, Jovaisa et al. found that administration of subanesthetic ketamine (0.5 mg/kg/h) in patients undergoing rapid opioid antagonist induction under general anesthesia suppressed precipitated withdrawal with no significant difference in abstinence at 4 months (22). In Iran, Mansoori et al. randomized 66 patients with comorbid MDD and opioid use disorder to ketamine or buprenorphine as adjunct to standard treatment. Anxiety and opioid craving improved in both arms, though along distinct trajectories: ketamine produced substantial anxiety reduction within hours, whereas buprenorphine yielded more gradual but sustained improvement in anxiety and craving (23). A 2026 single-blind RCT (n=68) of ketamine-assisted Mindfulness-Oriented Recovery Enhancement (MORE) found that patients on buprenorphine for the treatment of OUD who were randomized to MORE + ketamine-assisted psychotherapy reported less drug use and craving compared to MORE alone (24).

Although risks exist, including cardiorespiratory distress, ketamine-induced cystitis, ketamine use disorder, and long-term cognitive decline and prolonged dissociation (25, 26), they may be mitigated through supervised administration, avoidance of co-administered CNS depressants and opioids, lowest-effective dosing, and structured monitoring analogous to FDA REMS for esketamine (27). Ongoing work includes a Phase 2 KetaMORE extension and a trial of early ketamine to improve methadone retention in OUD with comorbid depression (28). Key limitations include that the current evidence base lacks generalizability as the foundational studies by Krupitsky were conducted in Russia and did not enroll fentanyl-using populations. Thus, ketamine’s efficacy in contemporary OUD is less clear. Funding is a further barrier: given that IV ketamine is highly bioavailable, generic, and widely available, there may be less commercial incentive to support the large, rigorous trials needed to establish its role in OUD and OWS.

### Cannabinoids

The endocannabinoid system (ECS) regulates stress, pain, and inflammation. Endocannabinoids include anandamide (AEA) and 2-arachidonoylglycerol (2-AG), which modulate CB1 and CB2 cannabinoid G-protein coupled receptors. The ECS and opioid systems share signaling pathways for pain management, addiction, and reward. For instance, CB1 and opioid receptors are often co-localized in brain regions responsible for pain processing. The two systems facilitate reward and addiction pathways, particularly through the nucleus accumbens (29). Given this overlap, there is therapeutic rationale for targeting the ECS in treatment of OUD.

Prior studies have investigated dronabinol, a synthetic delta-9-tetrahydrocannabinol (THC) and partial cannabinoid agonist, for managing opioid withdrawal. In a double-blind RCT, Bisaga showed that in opioid-dependent patients undergoing inpatient rapid buprenorphine detoxification onto extended-release naltrexone, dronabinol 30 mg reduced opioid withdrawal symptoms during the acute withdrawal phase compared with placebo (30). Of note, these patients had prior experience smoking marijuana to avoid exposing participants naïve to THC effects. Lofwall et al. showed that patients with self-reported use of short-acting opioids (n=12) receiving 20 and 30 mg of dronabinol experienced modest suppression of withdrawal symptoms after abruptly stopping opioids (31).

Cannabidiol (CBD) may modulate opioid cravings and cues. CBD lacks the psychotropic effects of THC and does not bind directly to cannabinoid receptors. Rather, CBD acts through TRPv1 agonism, 5-HT1A agonism, GPR55 antagonism, and opioid receptor modulation (32). The mechanism of CBD’s effects at the receptor level is complex. Laprarie et al. demonstrated that CBD is a negative allosteric modulator of the CB1 receptor (33). In a series of molecular binding assays on rat cerebral cortex membrane homogenates, Kathmann et al. showed that CBD is a negative allosteric modulator of the mu-opioid and delta-opioid receptors (34). However, the effects seen by Kathmann occurred at very high concentrations of CBD, at levels far above expected for patients receiving CBD therapy. Therefore, the *in vivo* mechanism of CBD effects on opioid receptors are currently unclear.

Given these different pharmacologic mechanisms, it is important to distinguish studies done with CBD and THC. CBD may support OUD treatment by attenuating responses to drug-related cues and reducing risk of relapse. A double-blind RCT by Hurd and colleagues found that CBD (400 or 800 mg daily for 3 consecutive days) reduced cravings and anxiety induced by drug cues in drug-abstinent individuals with heroin use disorder (n=42 total, 15 assigned to placebo, 14 to 400 mg CBD for 3 consecutive days, 13 to 800 mg CBD for 3 consecutive days). Notably, these effects persisted for 7 days after the last CBD dose (35). Suzuki et al. performed a double-blind, randomized crossover trial in participants with OUD receiving buprenorphine or methadone (n=10), showing that a one-time 600 mg CBD dose was associated with significant decrease in cue-induced craving and attentional bias toward drug-related cues in patients on buprenorphine or methadone (36).

If CB1 agonists like dronabinol can reduce opioid withdrawal symptoms, such compounds could be studied as adjuncts to manage withdrawal and improve induction success rates. Future studies focus on investigating whether CBD can reduce opioid relapse by attenuating environmental cue responses among individuals taking MOUD. Building on the cue-reactivity signal, Hurd and colleagues are conducting follow-on adjunctive-CBD trials in patients maintained on methadone or buprenorphine, including a Phase 3 study (NCT06940674) (37), evaluating whether CBD reduces illicit opioid use, and a Phase 2 study (NCT06206291) (38) of twice-daily CBD on cue-induced craving and anxiety. Of note, Cannabidiol (tradename Epidiolex) is approved by the FDA for the treatment of seizures for patients with Lennox-Gastaut syndrome, Draxvet syndrome, and tuberous sclerosis complex (39) and was removed from substance scheduling in 2020 (40). Risks are generally mild. CBD is well tolerated; principal concerns are dose-dependent diarrhea, somnolence, transaminase elevations (particularly with concomitant valproate), and clinically relevant CYP interactions (e.g., with clobazam). THC-containing agents such as dronabinol carry additional risks of tachycardia, anxiety/dysphoria, cognitive impairment, and potential for cannabis use disorder. Key literature limitations specific to cannabinoids include variability in cannabinoid formulations and dosing.

### Psychedelics

Once associated with the counterculture movement and restricted from research as Schedule I substances, psychedelics have seen a recent resurgence of interest in the treatment of SUDs due to their ability to induce neuroplastic changes in addiction circuits and create psychologically meaningful experiences that may motivate behavior change (41). Ibogaine has the largest evidence base for OUD among psychedelics, though the evidence remains limited and largely predates the fentanyl era. The only placebo-controlled RCT evaluated noribogaine, its active metabolite, as a safety study, in patients with OUD stabilized on methadone, demonstrating dose-dependent QTc prolongation; the trial was not designed to evaluate withdrawal symptoms, and the secondary endpoint did not reach significance (42). Several observational studies have broadly reported short-term reductions in withdrawal symptoms and longer-term benefits, including sustained abstinence, in patients using prescription opioids and heroin (43–46). Despite these signals, ibogaine is controversial due to increased risk of QTc prolongation and fatal arrhythmias. Several fatalities associated with ibogaine use have been documented in medically unsupervised settings, suggesting that careful prescreening and cardiac monitoring may reduce this risk. Emerging analogues such as tabernanthalog (47) and oxa-iboga compounds (48) show preclinical reductions in drug-seeking behavior with lower arrhythmia risk, and dosing strategies, such as co-administration with intravenous magnesium, have potential as future avenues for ibogaine research (49).

Outside ibogaine, the evidence is sparse. A review by Weleff et al. found only one LSD study and one ayahuasca study meeting inclusion criteria, with no completed psilocybin or MDMA trials for OUD (43). A randomized control trial (n=74) of LSD in incarcerated individuals who used heroin by Savage and McCabe found higher verified abstinence at 12 months in patients undergoing LSD-assisted residential treatment versus an outpatient control (25% vs. 2%) (50), though the trial was unblinded with substantially different therapeutic environments between groups. In a small observational study of ayahuasca-assisted group therapy in patients with varied substance use disorders (n=12), Thomas et al. observed improvements in hopefulness and mindfulness, but no change in opioid use in one user (51). In stark contrast to alcohol and nicotine use disorder, psilocybin remains almost entirely unstudied in OUD, with only a single safety report of psilocybin co-administered with buprenorphine in two patients (52). To our knowledge, several trials are now underway. For psilocybin, NYU is running a NIDA-funded Phase 2 trial as an adjunct for methadone-maintained patients with ongoing illicit use (NCT06796062), with additional academic programs at Penn and the University of Wisconsin–Madison. For ibogaine, DemeRx’s noribogaine has received FDA clearance to begin the first U.S. trial of an ibogaine-class compound (53) and Texas has launched a state-funded consortium for FDA-regulated ibogaine trials in OUD (54).

Aside from the risks discussed with ibogaine, classic serotonergic psychedelics (psilocybin, LSD) carry low physiological risk but meaningful psychological risk, including transient anxiety or “bad trips,” and rare persistent perceptual or psychotic reactions. They are contraindicated in primary psychotic disorders and in patients on serotonergic psychotropics (55). Key limitations include Schedule I status, which constrains supply, funding, and trial conduct; the resulting predominance of small, frequently unblinded or observational studies, ethics around consenting to a hallucinogenic experience (56), and the profound functional unblinding produced by the subjective drug experience.

### Incretin-based therapies: GLP-1 RAs and dual GIP/GLP-1 RAs

GLP-1 and glucose-dependent insulinotropic polypeptide (GIP) are incretin hormones with pleiotropic effects across various organ systems, including centrally regulating food intake and satiety (57). Exendin-4, a GLP-1 RA originally isolated from the venom of the Gila monster (Heloderma suspectum), served as the basis for exenatide, the first synthetic GLP-1 receptor agonist developed for clinical use (58). Apart from well-known metabolic indications, GLP-1 RAs (e.g., exenatide, liraglutide, semaglutide) and dual GIP/GLP-1 receptor agonist tirzepatide have recently generated interest as addiction therapeutics, as GLP-1 and GIP receptors are expressed in the mesolimbic pathway, specifically the ventral tegmental area (VTA) and nucleus accumbens (NAc), which are key areas in dopaminergic reward and learning pathways (59, 60).

There are multiple preclinical studies investigating GLP-1 RA on opioid use behaviors. For example, Liraglutide decreased heroin self-administration in rodent models as well as cue-induced reinstatement in subjects with high baseline use (61). Furthermore, liraglutide decreased heroin-seeking in cue-induced extinction, drug-primed reinstatement, and stress induced relapse models (62). However there remains a notable lack of studies in humans, specifically RCTs. Much of the current evidence derives from retrospective observational studies in patients with comorbid metabolic disorder, which brings concerns for generalizability to a population without such medical conditions.

Qeadan et al. (63) analyzed retrospective cohort data from 503,747 patients with OUD across 136 U.S. health systems, and reported that GIP/GLP-1 RA prescription was associated with a lower rate of overdose (63). The trial did not specify type of GLP1-RA, only presence of a GLP1-RA prescription. In 2024, a target trial emulation cohort study with over 33,000 patients with comorbid type 2 diabetes and OUD similarly found semaglutide associated with lower risk of opioid overdose compared with other antidiabetic agents (e.g., insulin, metformin, etc.) (64). A nested case-control analysis of the All of Us program (>142,000 participants with type 2 diabetes or obesity) reported GLP-1 RA exposure associated with lower odds of incident substance use disorders across categories, including approximately 69% lower odds for OUD; among agents, semaglutide showed the strongest signal (65). Most recently, a Veterans Affairs cohort found initiation on GLP-1 RA versus SGLT-2 inhibitors associated with reduced incident OUD and, among patients with pre-existing SUD, reductions in overdose and substance-related mortality (66). Risks include gastrointestinal side effects (e.g., nausea, vomiting, diarrhea, constipation, delayed gastric emptying, appetite suppression), weight loss, and malnutrition, possibly even leading to Wernicke’s encephalopathy (67, 68). Further risks include medullary thyroid carcinoma, which remains a rodent-derived contraindication (69). Given that these patients may be socioeconomically vulnerable, appetite suppression and weight loss warrant concerns in patients with food insecurity. The evidence base around GLP-1 RAs is limited, as it has largely only examined subjects with a comorbid metabolic or diabetic disorder.

Adequately-powered randomized controlled trials are needed before efficacy can be established. Brenipatide (LY3537031), Eli Lilly’s investigational dual GIP/GLP-1 receptor agonist, is being evaluated for opioid use disorder, with results for a phase 2 trial for OUD anticipated in 2027-2028(NCT07420283). Suzuki et al. is conducting semaglutide placebo-controlled RCT in patients on MOUD with results anticipated in 2027(NCT06548490).

## Discussion

OUD remains a major public health crisis in the U.S. Even with evidence-based, lifesaving treatments for patients with OUD, gaps persist in treatment retention, accessibility, and tolerability. The immediate priority is not replacing MOUD but determining whether these agents can safely and effectively augment care. Efforts for novel treatments attempt to engage beyond the µ-opioid-receptor, exploring the broader circuitry including reward and incentive salience, stress, hyperkatifeia, neuroplasticity, learning, and comorbid mood/anxiety disorders. Despite differences in each class’s mechanism of action, one potential convergence is the mesolimbic reward circuitry, particularly on cue-reactivity (70). Early preclinical and clinical evidence suggests ketamine could mediate cue-responsive mesolimbic activity (71), CBD may attenuate cue-induced craving and attentional bias (35, 36), psychedelics potentially affect neuroplasticity in which cue-driven associations can be relearned (72), and GLP-1 RAs have been shown to suppress incentive motivational properties of cues in other substances (60). Consequently, cue-induced craving could serve as a shared mechanistic target and a potential common endpoint for future trials. Though promising, the evidence base clearly varies significantly across the four therapeutic classes and further research is needed to elucidate mechanism, safety, and efficacy.

The distribution of evidence across these classes is shaped substantially by drug scheduling, reflecting perceived risk and historical trends (**Table 1**). Ketamine, GLP-1 RAs, and CBD are FDA-approved for existing indications, clinically accessible, and have a larger body of evidence with randomized trials, off-label clinical experience, and large observational datasets. Psychedelics and ibogaine remain Schedule I - a classification that imposes restricted drug supply, constrained funding, and institutional barriers and has limited the evidence base largely to small, frequently unblinded or observational studies. Ibogaine has the least rigorous controlled evidence of the compounds reviewed, reflecting regulatory policy rather than lack of biological promise. Nevertheless, the safety concerns associated with ibogaine are substantial and warrant careful consideration. Notably, however, much of the existing safety signal derives from “underground” use, reflecting substantial patient demand and the limited availability of regulated alternatives. Regulated research access would permit both higher-quality characterization of efficacy and risk and the conduct of studies under medical supervision. A recent executive order in April 2026 allocates $50 million to states for psychedelic research and accelerated pathways through FDA approval (73). From this perspective, the regulatory pathways, funding, and infrastructure available to investigators are themselves determinants of the evidence base, and clarifying the role of these agents in OUD will depend on the capacity to study them under adequately monitored conditions rather than on current perceptions of risk alone. Scheduling reform alone, however, may not substitute for methodological rigor, as the ketamine and GLP-1 literatures demonstrate, as the American Psychiatric Association has called for an evidence-driven approach (74).

**Table 1:** Comparison of four emerging pharmacotherapy classes for opioid use disorder.

Class	Primary mechanism (beyond MOR)	Key efficacy signal	Principal safety/feasibility constraint	US regulatory status
Ketamine/NMDA antagonists	NMDA antagonism → glutamate surge with AMPA–BDNF–TrkB–mTOR neuroplasticity; downregulation of cue-responsive mesolimbic circuits; possible direct opioid-receptor activity	Dose-dependent abstinence at 1–2 yr; reduced withdrawal during antagonist induction; rapid craving and anxiety reduction	Dissociation, hemodynamic effects, respiratory depression in high doses, urologic toxicity, diversion, theoretical ketamine use disorder; requires monitored setting	Esketamine FDA-approved (TRD) under REMS; racemic IV off-label; **not approved for OUD**
Cannabinoids (CBD, dronabinol)	Endocannabinoid CB1/CB2 modulation; CBD acts non-CB1 via TRPV1, 5-HT1A, GPR55, and opioid-receptor modulation; shared reward and pain circuitry	CBD reduced cue-induced craving and anxiety (persisting ~7 days); dronabinol modestly suppressed withdrawal	Tachycardia, sedation and impaired cognitive effects, THC intoxication; formulation and dosing heterogeneity	CBD (Epidiolex) Schedule V, approved for seizures only; medical cannabis partially rescheduled to Schedule III (April 2026); **not approved for OUD**
Psychedelics (ibogaine-focused)	Multimodal serotonergic (5-HT2A) and other actions; ibogaine acts at NMDA, nicotinic, opioid, sigma, and monoamine-transporter sites; purported interruption of drug-seeking via plasticity	Observational short-term withdrawal reduction and sustained abstinence (ibogaine); LSD 12-month abstinence 25% vs 5% (unblinded)	Ibogaine QTc prolongation and fatal arrhythmia, with documented deaths; dissociation, ataxia, nausea, vomiting, supervised administration required; analogues in development. Unclear if risk for substance use liability.	Schedule I; 2026 executive order created a Right-to-Try pathway including ibogaine; DemeRx noribogaine cleared for first US ibogaine-derivative trial; **not approved**
GLP-1/dual GIP–GLP-1 agonists	GLP-1 receptors in VTA, NAc, and striatum modulate dopaminergic reward; reduced cue-induced reinstatement; HPA-axis and stress modulation; possible glutamatergic effects	~40% lower overdose rate; up to ~69% lower odds of incident OUD (semaglutide largest effect), in metabolic/diabetic populations	GI intolerance (nausea, vomiting), titration burden; pancreatitis and C-cell signals; weight loss or malnutrition in low-weight or food-insecure patients	Approved for type 2 diabetes, obesity, and cardiovascular risk; **no SUD/OUD indication** (off-label); unscheduled

MOR, mu-opioid receptor; RCT, randomized controlled trial; TRD, treatment-resistant depression; REMS, Risk Evaluation and Mitigation Strategy; VTA, ventral tegmental area; NAc, nucleus accumbens; GI, gastrointestinal; HPA, hypothalamic–pituitary–adrenal.

Bolded terms indicate current status of FDA approval for OUD treatment.

For these therapies, limitations are important to consider. First, there exists an expectancy bias despite blinding, where perceptible drug effects such as dissociation, psychoactivity associated with psychedelics, ketamine, and cannabinoids, and gastrointestinal side effects associated with GLP1s unblind *a priori* allocation. Furthermore, there is a shifting phenotype of opioid use disorder, as reflected in evolving chemical potency and users, such that foundation trials precede the fentanyl-dominant era, so their current applicability is unclear (20, 21). In terms of generalizability, there may be sample skew and underrepresentation of minorities in novel therapies, especially given previous breaches of trust (e.g., Tuskegee). Furthermore, this narrative review demonstrates that existing research varies significantly in terms of endpoint, dosing, type of drug, patient population, and length of follow-up and requires standardization.

Addressing these limitations requires a two-pronged approach: coordinated research methodology and policy reform. Trials should incorporate active placebo or comparators approximating the agent’s effects, formally assess and report blinding integrity rather than assume it, and prioritize blinded, objective endpoints over self-report. Given small samples and high placebo response, the sequential parallel comparison design, which re-randomizes placebo non-responders and pools across phases, offers improved efficiency (75). Concurrently, streamlining approval pathways, funding, and trial infrastructure for Schedule I agents, but also all therapeutics under study, is a precondition for this rigor in OUD: regulatory reform enables better studies, and better studies inform sound regulation.
